# Temporal Changes in Extracellular Vesicle Hemostatic Protein Composition Predict Favourable Left Ventricular Remodeling after Acute Myocardial Infarction

**DOI:** 10.3390/ijms24010327

**Published:** 2022-12-25

**Authors:** Xiong Chang Lim, Chenyuan Huang, Siti Maryam J. M. Yatim, Suet Yen Chong, Sock Hwee Tan, Xiaoxun Yang, Caryn L. Heldt, Jodi Pedersen, Michael Talanker, Harshvardhan Modh, Matthias G. Wacker, Giorgia Pastorin, Siew Pang Chan, A. Mark Richards, Chris J. Charles, Mark Y. Chan, Jiong-Wei Wang

**Affiliations:** 1 Department of Surgery, Yong Loo Lin School of Medicine, National University of Singapore, Singapore 119228, Singapore; 2 Cardiovascular Research Institute (CVRI), National University Heart Centre Singapore (NUHCS), Singapore 117599, Singapore; 3 Department of Medicine, Yong Loo Lin School of Medicine, National University of Singapore, Singapore 119228, Singapore; 4 Department of Chemical Engineering, Michigan Technological University, Houghton, MI 49931, USA; 5 Department of Pharmacy, Faculty of Science, National University of Singapore, Singapore 119228, Singapore; 6 Department of Medicine, Christchurch Heart Institute, University of Otago, Christchurch 8140, New Zealand; 7 Department of Physiology, Yong Loo Lin School of Medicine, National University of Singapore, Singapore 119228, Singapore; 8 Nanomedicine Translational Research Programme, Centre for NanoMedicine, Yong Loo Lin School of Medicine, National University of Singapore, Singapore 117609, Singapore

**Keywords:** extracellular vesicles, coagulation, fibrinolysis, acute myocardial infarction, ventricular remodeling, heart failure

## Abstract

The subset of plasma extracellular vesicles (EVs) that coprecipitate with low-density lipoprotein (LDL-EVs) carry coagulation and fibrinolysis pathway proteins as cargo. We investigated the association between LDL-EV hemostatic/fibrinolysis protein ratios and post-acute myocardial infarction (post-AMI) left ventricular (LV) remodeling which precedes heart failure. Protein concentrations of von Willebrand factor (VWF), SerpinC1 and plasminogen were determined in LDL-EVs extracted from plasma samples obtained at baseline (within 72 h post-AMI), 1 month and 6 months post-AMI from 198 patients. Patients were categorized as exhibiting adverse (n = 98) or reverse (n = 100) LV remodeling based on changes in LV end-systolic volume (increased or decreased ≥15) over a 6-month period. Multiple level longitudinal data analysis with structural equation (ML-SEM) model was used to assess predictive value for LV remodeling independent of baseline differences. At baseline, protein levels of VWF, SerpinC1 and plasminogen in LDL-EVs did not differ between patients with adverse versus reverse LV remodeling. At 1 month post-AMI, protein levels of VWF and SerpinC1 decreased whilst plasminogen increased in patients with adverse LV remodeling. In contrast, VWF and plasminogen decreased whilst SerpinC1 remained unchanged in patients with reverse LV remodeling. Overall, compared with patients with adverse LV remodeling, higher levels of SerpinC1 and VWF but lower levels of plasminogen resulted in higher ratios of VWF:Plasminogen and SerpinC1:Plasminogen at both 1 month and 6 months post-AMI in patients with reverse LV remodeling. More importantly, ratios VWF:Plasminogen (AUC = 0.674) and SerpinC1:Plasminogen (AUC = 0.712) displayed markedly better prognostic power than NT-proBNP (AUC = 0.384), troponin-I (AUC = 0.467) or troponin-T (AUC = 0.389) (*p* < 0.001) to predict reverse LV remodeling post-AMI. Temporal changes in the ratios of coagulation to fibrinolysis pathway proteins in LDL-EVs outperform current standard plasma biomarkers in predicting post-AMI reverse LV remodeling. Our findings may provide clinical cues to uncover the cellular mechanisms underpinning post-AMI reverse LV remodeling.

## 1. Introduction

Advances in revascularization and adjunctive pharmacotherapies have reduced the mortality from acute myocardial infarction (AMI) over recent decades. However, heart failure (HF) secondary to post-AMI adverse (or maladaptive) left ventricular (LV) remodeling remains a leading cause of morbidity and mortality worldwide [1]. The macroscopic manifestations of post-AMI adverse LV remodeling are characterized by gradual geometrical changes including peri-infarct and more remote wall thinning, increased ventricular volumes and mass, conversion from an ovoid to a more spherical architecture and consequent deterioration in function. Activation of inflammatory and coagulant pathways, cardiac fibrosis and microvascular obstruction promote adverse LV remodeling [2,3]. Conversely, reduction in LV volumes, defined as “reverse” LV remodeling, occurs in a proportion of post-infarct cases and is associated with improved cardiac function which, alongside pharmacotherapy targeting neurohormonal pathways, results in improved outcome and reduced progression to HF [4,5]. Change in LV end systolic volume (LVESV; applying a cutoff of ±15%) at six months after MI is a widely accepted echocardiographic measure to define adverse (−) or reverse (+) LV remodeling [5,6]. Given the relatively high interobserver variability in echocardiographic measurement of cardiac volumes, combining changes in LVESV with circulating biological markers associated with the pathophysiological process of LV remodeling may improve the accuracy of risk stratification to better guide clinical management and ensure patients at high-risk of adverse LV remodeling receive more timely and effective intervention.

Extracellular vesicles (EVs) are lipid bilayer membrane vesicles containing biological material (proteins, lipids, RNAs and microRNAs) derived from the cell of origin that reflect the prevailing physiological and/or pathological conditions at the time of EV packaging and secretion [7]. Thus, plasma EVs have emerged as a potential source of biomarkers for cardiovascular diseases [8,9,10,11,12,13]. EVs released from various cardiac cells post-AMI may contribute to LV remodeling by mediating intercellular communication [14,15,16,17,18]. Furthermore, plasma EVs are markedly increased following AMI [19,20,21]. EVs from AMI patients are correlated with myocardial microvascular obstruction and cause endothelial dysfunction in vitro [15,21]. Plasma EVs from AMI patients harbor hemostatic proteins including tissue factor and Serpin F2 [20,22], suggesting possible involvement of thrombogenic EVs in post-AMI remodeling. Previous reports have provided informative cross-sectional “snapshots” of circulating EVs in AMI patients [14,15,19,20]. However, there are few longitudinal studies investigating temporal change in plasma EVs during the progression of well documented post-AMI LV remodeling.

After precipitating low-density lipoprotein (LDL) from plasma, we recently identified a subset of coprecipitated procoagulant EVs (LDL-EVs) containing several hemostatic proteins [23]. We now explore whether temporal changes in LDL-EV related hemostatic proteins, and the ratios between procoagulant and fibrinolytic proteins in particular, are associated with post-AMI LV remodeling in a clinical cohort (primary outcome), given their potential contrasting roles in post-MI healing and scaring. The secondary outcomes of this study are (1) clinical correlations of LDL-EV protein ratios in patients with adverse and reverse LV remodeling with selected clinical variables and (2) prognostic value of these protein ratios for predicting post-AMI LV remodeling.

## 2. Methods

### 2.1. Study Design and Population

**Figure 1:**
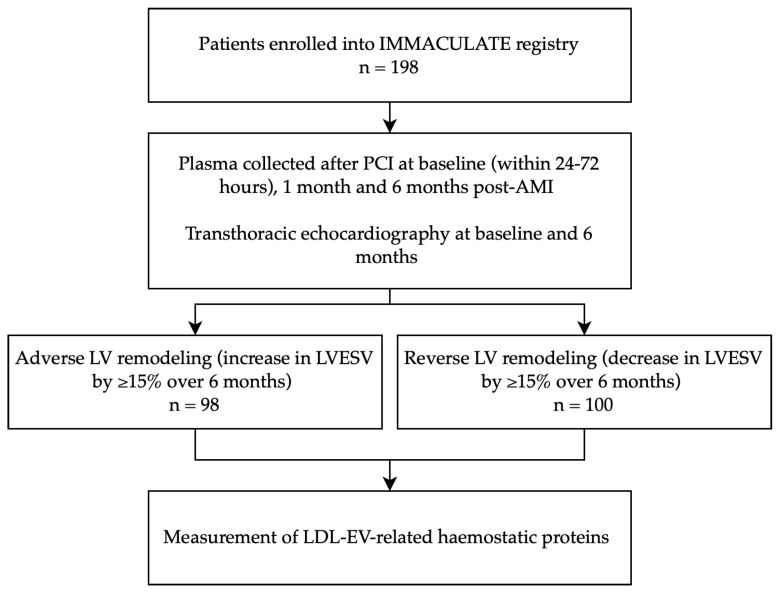
Study design flowchart. Abbreviations: PCI, percutaneous coronary intervention; LVESV, left ventricular end systolic volume.

The present study is a nested case-control study matching patients with adverse LV remodeling to patients with reverse LV remodeling from the IMMACULATE registry study (Figure 1) [24,25]. The IMMACULATE registry was a multicenter study and the inclusion criteria for this study are as follows: history of typical ischemic chest pain lasting more than 30 min, electrocardiographic changes, cardiac troponin levels above the 99th percentile upper reference limit and angiographic findings of more than 50% occlusion in one or more coronary arteries. Patients above 85 years of age, with valvular heart disease, cardiogenic shock, malignancy, renal impairment (eGFR < 15 mL/min/1.73 m^2^), liver impairment, anemia, HIV, hepatitis B or hepatitis C were excluded.

Blood samples were collected into sodium-citrate tubes at three timepoints, namely within 24–72 h after percutaneous coronary intervention (PCI) (baseline), 1 month and 6 months post-AMI. Samples were centrifuged and aliquoted plasma was stored at −80 °C prior to assay for N-terminal pro brain natriuretic peptide (NT-proBNP), high-sensitivity troponin T (hsTnT), high-sensitivity troponin I (hsTnI) and underwent isolation of LDL-EVs. Patients underwent transthoracic echocardiography for assessment of cardiac structure and function at baseline and 6 months. Based on the change in LV end-systolic volume (LVESV) over 6 months [5,6], we selected two groups of patients: adverse LV remodeling (n = 98) included those with more than 15% increase in LVESV, and reverse LV remodeling (n = 100) included those with more than 15% decrease in LVESV. Patients were followed up for major adverse cardiac and cerebrovascular events (MACCEs), i.e., cardiovascular death, HF, recurrent MI and ischemic stroke, for up to 2 years. The diagnosis of HF was adjudicated according to the European Society of Cardiology criteria by two clinicians (an emergency department specialist and a cardiologist) [1]. All participants provided written informed consent. The study was approved by the National University of Singapore Institutional Research Board (NHG DSRB Ref: 2015/01156) and was conducted in accordance with the principles of the Declaration of Helsinki.

### 2.2. Isolation of LDL-EVs

Plasma LDL-EVs were isolated as described previously [23]. Briefly, 6.5% (*w*/*v*) dextran sulphate (DS) and 2 M Manganese (II) chloride (MnCl_2_) (Sigma-Aldrich, St. Louis, MO, USA) stock solutions were prepared. Sequentially, DS stock (1:125, *v*/*v*) and MnCl2 (1:40, *v*/*v*) were added into 125 µL plasma. The samples were mixed and centrifuged at 4800 *g* for 10 min at 4 °C. The pellets (LDL-EV fractions) were either resuspended for characterization, or lysed in 125 µL lysis buffer (Roche #04719956001, Basel, Switzerland) for protein analysis. Samples were stored at −80 °C until analysis.

### 2.3. Characterization of LDL-EVs 

LDL-EVs were resuspended in filtered phosphate-buffered saline (PBS) and size and concentration measured by nanoparticle tracking analysis (NTA) using NanoSight NS300^TM^ (Malvern Instruments, Malvern, UK). All acquisitions were carried out at a camera level setting of 11, 13 or 14, and five videos of 60 s duration were recorded for each sample at 25 °C. The captured NTA videos were analyzed with a detection threshold of 5–7. To determine the surface charge of LDL-EVs, zeta potential was measured in duplicates at 25 °C using Litesizer 500 (Anton-Paar, Graz, Austria).

For transmission electron microscopy (TEM), 20 µL of isolated LDL-EVs were fixed with 2.5% glutaraldehyde for 1 h at 4 °C and then mounted on a Formvar Film 200 mesh, CU, FF200-Cu grid for 30 min. After 1 min staining with 5% of gadolinium triacetate, images were taken at room temperature using the FEI Tecnai G2 Spirit transmission electron microscope (FEI Company, Hillsboro, OR, USA).

### 2.4. Quantitative Protein Assay

Protein concentrations were determined using a beads-based multiplex-immunoassay as previously described [23]. Briefly, the beads (Luminex MagPlex-C Microspheres, MC100-xx, Austin, TX, USA) were coupled with selected antibodies to form a bead-capture antibody complex. Samples were incubated with the antibody complex and the corresponding biotinylated detection antibodies. Streptavidin-phycoerythrin (SA-PE, BD Bioscience #554061, San Jose, CA, USA) was added for quantification of captured proteins. Standard dilution curves for homologous recombinant proteins were prepared for calculation of protein concentration. Measurement and data analysis were performed using the Bio-Plex^®^ 200 Systems (Bio-Rad #171-000201, Hercules, CA, USA). The antibodies and recombinant proteins were as follows: for detection of VWF we used recombinant human VWF protein (Factor VIII free, Fitzgerald #30C-CP4003U, Fitzgerald Industries International, Acton, MA, USA), anti-human VWF (Fitzgerald #70R-10589, Fitzgerald Industries International, Acton, MA, USA), and biotinylated anti-human VWF (Fitzgerald #60R-1019, Fitzgerald Industries International, Acton, MA, USA); for detection of SerpinC1, anti-thrombin III antibody (NOVUS Biologicals #NBP1-05149, Littleton, CO, USA), human SerpinC1 biotinylated affinity purified antibody (R&D Systems #BAF1267, Minneapolis, MN, USA) and recombinant human SerpinC1 (R&D Systems #1267-PI-010, Minneapolis, MN, USA); for detection of plasminogen, anti-human plasminogen (NOVUS Biologicals NB120-10176, Littleton, CO, USA), biotinylated anti-human plasminogen (NOVUS Biologicals, NB120-10177, Littleton, CO, USA), and recombinant human plasminogen (R&D system,1939-SE-200, Minneapolis, MN, USA); and for detection of SerpinF2, anti-human SerpinF2 (R&D Systems #MAB1470, Minneapolis, MN, USA), biotinylated anti-human SerpinF2 (R&D Systems #BAF1470, Minneapolis, MN, USA) and recombinant human SerpinF2 (R&D Systems #1470-PI-010, Minneapolis, MN, USA).

### 2.5. Statistical Analysis 

The data are presented as means ± standard deviation (SD), median ± interquartile range (IQR) or percentage, depending on their nature. Exploratory analyses were performed with Student’s *t*-test or Mann–Whitney U test. The differences between baseline and follow-up measurements were ascertained with the Wilcoxon signed-ranked test. Confirmatory analysis was performed with the multilevel structural equation models (ML-SEM) and ANCOVA to ascertain if there was a significant difference in LDL-EVs protein levels between patients with adverse and reverse LV remodeling while adjusting for baseline factors including age, gender, ethnicity, diabetes, hypertension, dyslipidemia, lipid profiles and medications. This model was proposed in view of the longitudinal design and the nature of research questions. Receiver operating characteristic (ROC) curve analysis was employed to investigate the utility of each LDL-EV protein ratio and their combination with cardiac markers and clinical variables for diagnosis of reverse LV remodeling. Kaplan–Meier survival curves and Mantel–Cox log-rank test were used to visually assess LV remodeling differences in time to rehospitalization for HF. All statistical tests were conducted at 5% level of significance, with SPSS 25.0 (IBM Corp., Armonk, NY, USA) and Stata MP 16.0 (Stata Corp., College Station, TX, USA).

## 3. Results

### 3.1. Study Population

Baseline characteristics of the study population are summarized in Table 1. The 198 patients comprised 98 patients with adverse LV remodeling and 100 patients with reverse LV remodeling defined by ±15% change in LVESV over the first 6 months post-AMI (Figure 1). There was no significant difference in age, sex and ethnicity between the two groups of patients. The two groups of patients also had similar medical history, diagnoses, current medications, and baseline levels of plasma lipids. More patients with adverse LV remodeling were using warfarin (11.2% vs. 2.0%; *p* = 0.009).

### 3.2. Plasma Levels of Standard Cardiac Markers

Plasma levels of NT-proBNP, hsTnT and hsTnI decreased over 6 months after AMI in both groups (Table 2). Compared to patients with reverse LV remodeling, patients with adverse LV remodeling had higher plasma levels of hsTnT and hsTnI at baseline, and higher plasma levels of NT-proBNP and hsTnT at 1 month and 6 months.

### 3.3. Characteristics of Plasma LDL-EVs

To investigate the association of plasma EVs with post-AMI LV remodeling, we firstly characterized the LDL-EVs isolated from plasma at baseline by TEM, NTA and a Litesizer 500. As shown in Supplementary Materials Figure S1, LDL-EVs from patients with adverse and reverse LV remodeling showed similar size, surface charge and plasma concentrations.

### 3.4. Temporal Changes of Hemostatic Protein Levels in LDL-EVs in Post-AMI LV Remodeling

**Figure 2:**
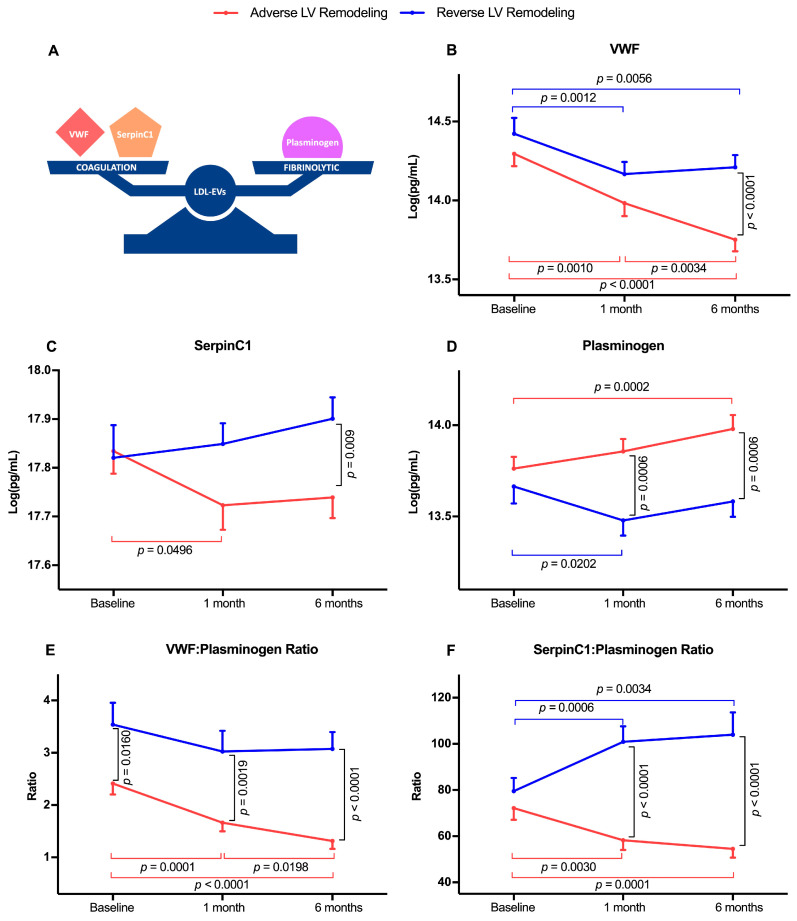
Temporal changes in LDL-EV hemostatic protein composition in post-AMI patients with adverse and reverse LV remodeling. Coagulation proteins (VWF, SerpinC1) and fibrinolytic protein (plasminogen) levels and their ratios (VWF:Plasminogen, SerpinC1:Plasmingen) in LDL-EVs of 198 post-AMI patients at baseline and after 1 and 6 month follow-up. (**A**) A diagram illustrating the studied hemostatic proteins in LDL-EVs. (**B**–**F**) Differences between baseline and follow-up measurements were established by Wilcoxon signed-ranked test (horizontal statistical bar). Differences in the three protein levels and the protein ratios between patients with adverse LV remodeling and reverse LV remodeling were established by Mann–Whitney U test (vertical statistical bar). Data are presented as mean ± SEM.

We next evaluated the temporal changes of three LDL-EV-related hemostatic proteins (Figure 2A), including VWF, SerpinC1 and plasminogen, and their associations with post-AMI LV remodeling. VWF levels declined progressively over 6 months in patients with adverse LV remodeling but only declined within the first month post-AMI in patients with reverse LV remodeling (Figure 2B). SerpinC1 levels declined within the first month and remained lower at 6 months post-AMI in patients with adverse LV remodeling but did not change in patients with reverse LV remodeling (Figure 2C). Plasminogen levels increased within the first month and remained higher at 6 months post-AMI in patients with adverse LV remodeling, then decreased within the first month and remained lower at 6 months post-AMI in patients with reverse LV remodeling (Figure 2D). When analyzed at individual timepoints, in comparison to patients with reverse LV remodeling, VWF levels at 6 months and SerpinC1 levels at both 1 month and 6 months post-AMI were lower and plasminogen levels at both 1 month and 6 months post-AMI were higher in patients with adverse LV remodeling.

Given the alteration in the balance between coagulation-related (VWF and SerpinC1) and fibrinolysis-related (plasminogen) proteins in post-AMI LV remodeling, we further evaluated the change of the ratios between coagulation proteins and plasminogen in post-AMI patients. VWF:Plasminogen ratio declined progressively over 6 months in patients with adverse LV remodeling but remained unchanged in patients with reverse LV remodeling (Figure 2E). SerpinC1:Plasminogen ratio declined within the first month and remained lower at 6 months post-AMI than at baseline in patients with adverse LV remodeling (Figure 2F). In contrast, the SerpinC1:Plasminogen ratio increased within the first month and remained significantly higher at 6 months post-AMI than that at baseline in patients with reverse LV remodeling. Compared to that in patients with reverse LV remodeling, both VWF:Plasminogen and SerpinC1:Plasminogen ratio were markedly lower at both 1 month and 6 months post-AMI in patients with adverse LV remodeling.

### 3.5. ML-SEM Modeling of LDL-EV Protein Ratios and Post-AMI LV Remodeling

To evaluate whether LDL-EV protein ratios have predictive value for LV remodeling independent of baseline differences, we performed statistical analysis with ML-SEM modeling that allows potential interactions between variables to be assessed (Table 3). Overall, patients with reverse LV remodeling had markedly higher ratios of VWF:Plasminogen and SerpinC1:Plasminogen than patients with adverse LV remodeling during the 6 month follow-up. The differences in these two ratios of LDL-EV proteins between patients with reverse versus adverse LV remodeling remained significant after adjusting for age, gender, ethnicity, medications, lipid profile and other cardiovascular disease risk factors.

Post hoc analysis with ANCOVA indicated the significant difference in ratios of VWF:Plasminogen and SerpinC1:Plasminogen between patients with reverse and adverse LV remodeling was apparent at 1 month, and remained significant at 6 months post-AMI.

### 3.6. Clinical Correlations of LDL-EV Proteins

ML-SEM with backward elimination was performed to identify clinical correlations of LDL-EV protein ratios in patients with adverse and reverse LV remodeling. A total of 14 clinical variables, including age, gender, ethnicity, comorbidity, cardiovascular disease risk factors and medications were examined (Table 3). VWF:Plasminogen ratio was significantly associated with age, LDL cholesterol, aspirin and statin treatment, whereas SerpinC1:Plasminogen ratio was associated with gender and Malay race. More importantly, both ratios of VWF:Plasminogen and SerpinC1:Plasminogen were associated with reverse LV remodeling in post-AMI patients, independent of other cardiovascular disease risk factors.

### 3.7. LDL-EV Protein Levels as a Predictor for Post-AMI LV Remodeling and Heart Failure

**Figure 3:**
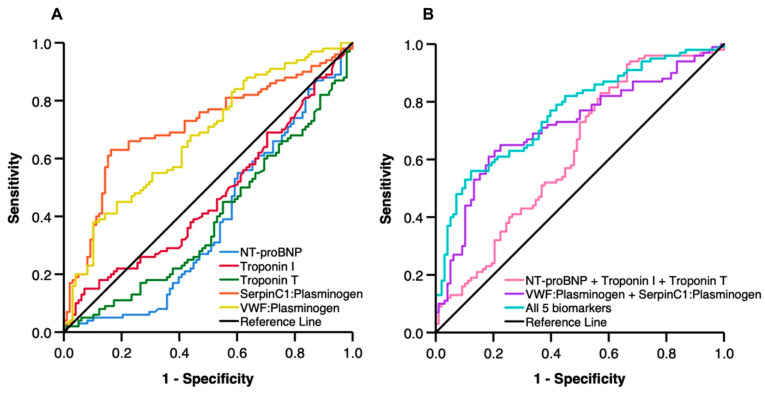
Receiver operating characteristic (ROC) analysis for identifying post-AMI patients with reverse LV remodeling at 1 month post-AMI. (**A**) ROC analysis for individual cardiac injury markers and coagulation/fibrinolysis protein ratios at 1 month post-AMI. (**B**) ROC analysis for combination of the LDL-EV protein ratios and three cardiac injury markers at 1 month post-AMI.

To test the prognostic value of the two coagulation/fibrinolysis protein ratios for predicting post-AMI LV remodeling, we compared their AUCs with the AUCs of three standard cardiac injury markers at 1 month post-AMI (Table 4 and Figure 3A). As expected, the low AUCs of NT-proBNP (0.384), hsTnI (0.467) and hsTnT (0.389) showed the poor power of standard cardiac injury markers to distinguish reverse LV remodeling from adverse LV remodeling. Interestingly, VWF:Plasminogen and SerpinC1:Plasminogen ratios displayed reasonably good prognostic power to predict reverse LV remodeling after AMI, with AUCs of 0.674 and 0.712, respectively. As analyzed by Delong’s method [26], the AUC of VWF:Plasminogen ratio was markedly higher than the AUCs of NT-proBNP (Z = 5.281, *p* < 0.001), hsTnI (Z = 3.604, *p* < 0.001) and hsTnT (Z = 5.215, *p* < 0.001). Similarly, the AUC of SerpinC1:Plasminogen ration was higher than the AUCs of NT-proBNP (Z = 6.130, *p* < 0.001), hsTnI (Z = 4.350, *p* < 0.001) and hsTnT (Z = 5.668, *p* < 0.001). These results suggest that the two ratios between coagulation and fibrinolysis proteins outperform the standard cardiac injury markers for predicting post-AMI LV remodeling.

**Figure 4:**
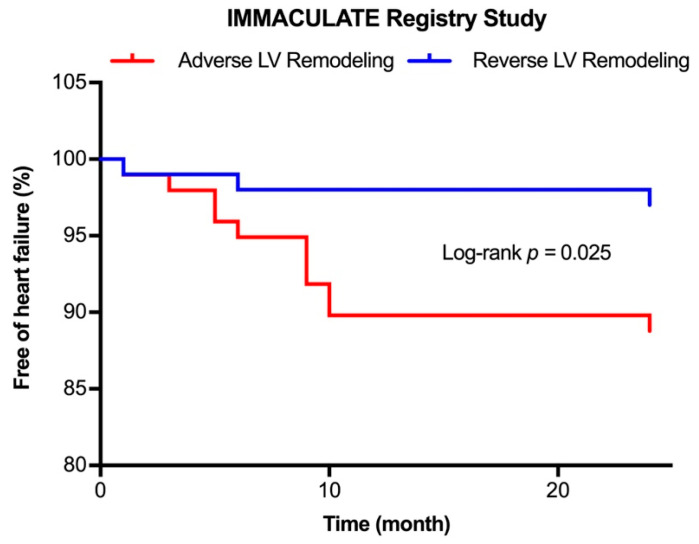
Incidence of heart failure during follow-up. Kaplan–Meier analysis for rehospitalization due to heart failure stratified by post-AMI LV remodeling.

Next, we assessed the performance of the combination of the LDL-EV protein ratios and three cardiac injury markers for prediction of post-AMI LV remodeling (Table 4 and Figure 3B). The receiver-operator characteristic (ROC) curves were compared among the 3 candidate prognostic biomarkers: (1) cardiac injury panel (NT-proBNP + hsTnI + hsTnT); (2) LDL-EV ratios (VWF:Plasminogen + SerpinC1:Plasminogen); and (3) the LDL-EV protein ratios combined with the cardiac injury panel. There was no statistically significant difference between the AUC of LDL-EV ratios (0.717) and the AUC of cardiac injury panel (0.628). However, the AUC of the combination model (0.763) was significantly higher than the AUC of cardiac injury panel (Z = 3.152, *p* = 0.002). Furthermore, during a median follow-up of 2 years (interquartile range 1.9–2 years), 11 out of 98 patients with adverse LV remodeling (11.3%) vs. 3 out of 100 patients with reverse LV remodeling (3.0%) were diagnosed with HF (*p* = 0.025; Figure 4).

## 4. Discussion

Plasma EVs released from the injured heart may reflect a “snapshot” of the pathophysiology of the myocardium and can potentially be used as surrogate biomarkers predictive of LV remodeling. The highly regulated coagulation and fibrinolytic systems are important mediators of inflammation and play an important role in tissue remodeling. We report the temporal changes of coagulation (VWF and SerpinC1) and fibrinolytic (plasminogen) protein levels as well as their ratios (VWF:Plasminogen and SerpinC1:Plasminogen) in plasma LDL-EVs obtained from post-AMI patients with adverse or reverse LV remodeling. We found that higher levels of coagulation proteins and lower levels of fibrinolytic protein in LDL-EVs were independently associated with reverse LV remodeling. Moreover, we demonstrate that temporal changes in the ratios between coagulation and fibrinolysis pathway proteins in LDL-EVs outperform current standard plasma biomarkers (NT-proBNP + hsTnI + hsTnT) in prediction of post-AMI reverse LV remodeling. To our knowledge, this is the first study that shows an association between hemostatic protein levels in EVs and post-AMI LV remodeling. The nature of our study means that the exact pathophysiological mechanisms and pathways underpinning this association cannot be defined from our existing data. We postulate that inflammation and coagulation play key roles in LV remodeling after AMI.

Cardiac remodeling results from several pathophysiological changes in response to cardiac injury. The higher baseline levels of plasma TnT in patients with post-AMI adverse LV remodeling (Table 2) suggest that more severe cardiac injury occurred in those patients, which might contribute to the adverse remodeling of LV thereafter [27]. Apart from cardiac injury, maladaptive mechanisms, including altered energy-related metabolic processes (excessive glucose oxidation but lowered free fatty acid oxidation), extracellular matrix, genetic expression, neurohumoral regulation, and cellular changes involving cardiomyocyte loss are observed as the heart remodels [27]. Cardiac remodeling was initially believed to be an irreversible process associated with decreased survival and worse outcomes [28]. However, in recent years, numerous therapeutic interventions have shown to slow adverse remodeling and/or to promote reverse remodeling [27]. Notably, LV assist device (LVAD) support has been associated with reverse ventricular remodeling through mechanical unloading, evidenced by improvements in blood pressure, cardiac output, and neurohormonal levels [29,30]. However, it is unclear if the perturbation of cardiac energy metabolism is restored in LV reverse remodeling. In addition, although different patterns of microRNA and long non-coding RNAs expression have been observed after LVAD support and might be useful in assessing the severity of HF [31,32,33], their potential as biomarkers of myocardial remodeling has yet to be elucidated. In this study, by analyzing the composition of hemostatic proteins in plasma LDL-EVs, we revealed that temporal changes in the ratios between coagulation and fibrinolysis pathway proteins were able to predict reverse LV remodeling as early as 1 month post-AMI. Compared to the categorization of adverse versus reverse LV remodeling based on echocardiography at 6 months post-AMI, our findings may help detect LV remodeling changes earlier, and early incorporation of targeted medical therapy and devices into the management plan can increase myocardial protection [34].

VWF, a pivotal coagulation protein that promotes thrombus formation via platelet adhesion and aggregation at the site of injury and acts as a carrier protein for coagulation factor VIII [35,36], is fundamentally involved in the pathologic mechanism responsible for AMI. Plasma VWF levels follow a typical time course during acute coronary syndrome, where they are elevated at 24 h and peak at 48 to 72 h [37]. In agreement with other studies, we observed the highest levels of VWF in LDL-EVs at 3 days post-AMI with VWF levels decreasing thereafter by 1 month in both patient groups. Interestingly, by 6 months, the adverse LV remodeling group displayed significantly lower VWF levels in LDL-EVs compared to the reverse LV remodeling group. This is seemingly counterintuitive since VWF is recognized as a mediator of inflammatory responses through promotion of leukocyte recruitment [38,39]. Although the mechanisms underlying our observation remain uncertain, we postulate that LDL-EVs carry sequestered VWF. As plasma VWF contributes to pro-inflammatory responses, it is plausible that the diminished VWF levels in LDL-EVs are caused by the homeostatic response in LV remodeling. SerpinC1, also known as antithrombin III, inhibits thrombin and other serine proteases, including Ixa, Xa, Xia and XIIa [40]. In addition, SerpinC1 inhibits inflammation via a coagulation-dependent or -independent pathway [41,42]. In our study, the higher levels of SerpinC1 in LDL-EVs from post-AMI patients with reverse LV remodeling may reflect its anti-inflammatory activity during cardiac repair. The counterpart of coagulation is fibrinolysis, a process that is highly regulated by multiple fibrinolytic proteases including plasminogen. Plasmin, the active form of plasminogen, is a key mediator of fibrinolysis activated by tissue-type plasminogen activator (tPA) and urokinase-type plasminogen activator (uPA) [43,44]. In our study, the elevated LDL-EV plasminogen levels in patients with adverse LV remodeling may increase the formation of toll-like receptor-4 (TLR-4), activating fibrin degradation products or activation of matrix metalloproteinases, which subsequently results in the release of matrix-bound growth factors [45,46]. Along with activation of PAR-1 by plasmin [47], these pro-inflammatory and pro-remodeling actions could possibly contribute to adverse LV remodeling in post-AMI patients.

The association of higher VWF and SerpinC1 levels and lower plasminogen levels in LDL-EVs with reverse LV remodeling were further evaluated with VWF:Plasminogen and SerpinC1:Plasminogen ratios. ML-SEM modeling of LDL-EV protein ratios and post-AMI LV remodeling revealed correlations between clinical variables and LDL-EV proteins ratios. The presence of several metabolic abnormalities, including hyperglycemia, hyperlipidemia, and hypercholesterolemia, in metabolic syndrome induce endothelial dysfunction by increasing reactive oxygen species and reducing nitric oxide [48]. As a result, biomarkers of endothelial dysfunction such as plasma VWF are elevated and associated with the presence of these deranged metabolic profiles [49]. In addition, LDL-EV VWF:Plasminogen levels were positively correlated with LDL cholesterol levels. We did not observe any significant difference in LDL-EV VWF in patients with and without hypertension despite previous studies reporting such associations for plasma VWF levels and hypertension [50]. Although a role for plasma VWF in maintaining hemostatic balance in the vasculature is well known, exactly what is reflected by the EV levels of VWF remains to be defined. Hence, the difference in association between levels of VWF with metabolic diseases depending on whether it is within the plasma or EVs may provide important mechanistic insights and warrant further discussion and research. Furthermore, we found decreased LDL-EV VWF:Plasminogen ratio in patients with statin treatment. These results are in line with our previously published report which shows positive association between rosuvastatin treatment and LDL-EV VWF and plasminogen levels [51].

LDL-EV SerpinC1:Plasminogen ratio was significantly lower in female than male patients. This suggests underlying gender-specific differences in pathophysiological mechanisms. Women tend to have less obstructive CAD and have relatively preserved LV function after MI [52,53]. Despite this, women have increased mortality and poorer prognosis compared to men. These studies suggest that abnormal coronary reactivity, microvascular dysfunction and distal microembolization are contributing factors to the underlying pathophysiology of angina and ACS in women [52,53,54]. This gender-specific difference may in part be due to the presence of varying reproductive hormone levels, involvement of autonomic nervous system adrenergic pathway, and the increased burden of pro-atherogenic risk factors in women [55]. These factors, among others may explain the differences in EV protein expression between men and women as demonstrated in our study.

### Limitations

First, for this proof-of-concept study, we selected two groups of patients with clear clinical phenotypes based on the 6 months post-AMI echocardiography; our findings need to be confirmed in other independent cohorts. Given the high percentage of male patients in both groups and the high incidence of MI in males [55,56], it is tempting to test the findings of this study in female patients. Secondly, LDL-EV protein levels prior to admission and at the point of AMI could yield additional insights. Unfortunately, plasma samples at those two time points were not available in this specific cohort. The relationship of free plasma concentrations of VWF, SerpinC1 and plasminogen to contemporaneous EV concentrations are not ascertained and may help elucidate the mechanisms underpinning the observed relationships of EV proteins to LV remodeling. In addition, we only characterized the size, surface charge and plasma concentrations of LDL-EVs at baseline because limited resources were available for this study, however, this limitation is unlikely to affect our conclusions, which are drawn from changes in LDL-EV hemostatic protein composition. Thirdly, the potential diagnostic/prognostic value of myriad other proteins in LDL-EVs in post-AMI LV remodeling remains to be explored. Finally, further preclinical studies are warranted to elucidate the pathophysiologic roles of LDL-EV proteins in post-AMI LV remodeling.

## 5. Conclusions

The ratios between coagulation and fibrinolysis proteins in plasma EVs outperform current standard plasma biomarkers in predicting post-AMI reverse LV remodeling. Temporal changes in LDL-EV hemostatic protein composition and their ratios highlight the potential of plasma LDL-EVs as a biomarker source for assessment of LV remodeling, which may be able to provide clinical insight for further investigation of the cellular mechanisms underlying post-AMI LV remodeling and to guide therapeutic interventions for prevention of HF.

## Figures and Tables

**Table 1:** Baseline Characteristics of the Study Population.

	Post-AMI		
	Adverse LV Remodeling	Reverse LV Remodeling	*p*-Value
	n = 98	n = 100	
**Demographic**			
Mean age (years)	55 (48–60)	54 (49–62)	0.963
Male (%)	93 (94.9)	93 (93.0)	0.576
Chinese (%)	54 (55.1)	56 (56.0)	0.130
Malay (%)	26 (26.5)	16 (16.0)	
Indian (%)	14 (14.3)	25 (25.0)	
Other (%)	4 (4.1)	3 (3.0)	
**Smoking status**			
Non-smoker (%)	28 (28.6)	32 (32.0)	0.869
Current smoker (%)	60 (61.2)	58 (58.0)	
Ex-smoker (%)	10 (10.2)	10 (10.0)	
**Medical history**			
Diabetes (%)	20 (20.4)	20 (20.0)	0.943
Dyslipidemia (%)	43 (43.9)	46 (46.0)	0.764
Hypertension (%)	44 (44.9)	40 (40.0)	0.486
**Lipid levels at baseline**			
Total cholesterol (mg/dL)	5.43 (1.4)	5.43 (1.3)	1.000
HDL cholesterol (mg/dL)	1.03 (0.9–1.3)	1.10 (0.9–1.3)	0.647
LDL cholesterol (mg/dL)	3.54 (1.2)	3.50 (1.3)	0.820
Triglycerides (mg/dL)	1.56 (1.2–2.4)	1.64 (1.1–2.4)	0.691
**Diagnoses**			
STEMI (%)	82 (83.7)	78 (78.0)	0.311
NSTEMI (%)	16 (16.3)	22 (22.0)	
**Medications**			
Aspirin (%)	95 (96.9)	98 (98.0)	0.634
P2Y12 inhibitor (%)	95 (96.9)	99 (99.0)	0.303
Statin (%)	96 (98.0)	98 (98.0)	0.984
Warfarin (%)	11 (11.2)	2 (2.0)	0.009
**Echocardiographic changes after 6 months**			
Change in LVEDV (%)	29.88 (18.3)	−12.26 (13.5)	<0.001
Change in LVESV (%)	29.43 (20.7–40.1)	−25.59 (−32.03–20.19)	<0.001
Change in EF (%)	−2.85 (−10.1–2.9)	16.43 (6.8–28.4)	<0.001

Footnotes for Table 1: Continuous data are presented as mean ± SD or median ± IQR, and statistical analysis of continuous data was performed using unpaired Student’s *t*-test or Mann–Whitney U test. Categorical variables are presented as %, with differences between the groups tested with χ^2^. Abbreviations: AMI, acute myocardial infarction; LV, left ventricular; HDL, high density lipoprotein; LDL, low density lipoprotein; STEMI, ST-elevation myocardial infarction; NSTEMI, non-ST-elevation myocardial infarction; LVEDV, left ventricular end diastolic volume; LVESV, left ventricular end systolic volume; EF, ejection fraction.

**Table 2:** Plasma levels of standard cardiac markers in study population.

Cardiac markers	Post-AMI					
	Adverse LV remodeling			Reverse LV remodeling		
	n = 98			n = 100		
	Baseline	1 month	6 months	Baseline	1 month	6 months
NT-proBNP	818.65 (397.50–1815.00)	614.00 (225.00–1285.00) **	205.85 (64.53–468.50) ***	789.95 (367.30–1202.00)	383.60 (143.25–697.50)	90.73 (38.78–192.30)
hsTnT	2807.00 (979.50–4406.00) ***	**15.68** (9.79–23.09) **	**10.37** (7.25–15.73) **	1578.50 (605.75–2632.00)	12.04 (7.83–16.62)	8.79 (5.99–12.55)
hsTnI	23219.40 (7359.60–43056.90) ***	12.70 (6.90–20.10)	7.45 (4.3–13.60)	9782.45 (3285.80–21938.75)	10.65 (5.90–19.35)	5.70 (3.25–10.55)

Footnotes for Table 2: Data are presented as median ± IQR. Significant differences between adverse and reverse remodeled groups at individual time-points are indicated as ** *p* ≤ 0.01; *** *p* ≤ 0.001. Abbreviation: AMI, acute myocardial infarction; LV, left ventricular; NT-proBNP, N-terminal pro-brain natriuretic peptide; hsTnT, high-sensitivity troponin T; hsTnI, high-sensitivity troponin I.

**Table 3:** ML-SEM modeling for LDL-EV proteins.

	Ratios of Coagulation Proteins and Fibrinolytic Protein in LDL-EVs					
	VWF:Plasminogen			SerpinC1:Plasminogen		
	Coefficient	*p*-Value	95% Cl	Coefficient	*p*-Value	95% Cl
**Demographic**						
Mean age	**0.054**	**<0.001**	**(0.025–0.083)**	−0.104	0.778	(−0.829–0.620)
Female	0.535	0.314	(−0.507–1.576)	**26.449**	**0.047**	**(0.340–52.558)**
Chinese	Ref	Ref	Ref	Ref	Ref	Ref
Malay	−0.171	0.582	(−0.779–0.437)	**−16.436**	**0.048**	**(−31.780–−1.093)**
Indian	0.017	0.957	(−0.602–0.635)	−13.950	0.092	(−29.302–1.402)
Other	0.342	0.645	(−1.112–1.80)	−25.197	0.179	(−61.956–11.563)
**Smoking status**						
Non-smoker	Ref	Ref	Ref	Ref	Ref	Ref
Current smoker	0.320	0.264	(−0.241–0.882)	10.312	0.148	(−3.652–24.277)
Ex-smoker	−0.126	0.780	(−1.008–0.757)	16.523	0.142	(−5.557–38.602)
**Medical history**						
Diabetes	−0.445	0.196	(−1.120–0.230)	1.367	0.875	(−15.632–18.365)
Dyslipidemia	0.445	0.115	(−0.109–1.000)	8.560	0.277	(−5.332–22.453)
Hypertension	−0.089	0.755	(−0.652–0.473)	2.808	0.694	(−11.192–16.809)
**Lipid levels at baseline**						
Total cholesterol	−0.457	0.061	(−0.935–0.022)	−2.235	0.715	(−14.228–9.759)
HDL cholesterol	0.119	0.816	(−0.882–1.120)	14.953	0.240	(−9.974–39.880)
LDL cholesterol	**0.565**	**0.035**	**(0.038–1.092)**	−1.813	0.788	(−15.041–11.415)
Triglycerides	0.061	0.319	(−0.059–0.181)	−0.527	0.731	(−3.531–2.477)
**Medication**						
Aspirin	**−2.397**	**0.004**	**(−4.008–−0.786)**	−4.483	0.828	(−44.926–35.959)
P2Y12 inhibitor	−1.970	0.054	(−3.977–0.037)	−15.068	0.560	(−65.690–35.553)
Statin	**−2.889**	**0.002**	**(−4.737–−1.040)**	29.643	0.213	(−17.047–76.332)
Warfarin	−0.774	0.172	(−1.886–0.338)	−12.604	0.378	(−40.601–15.393)
**Changes in protein levels**						
Baseline	**0.416**	**<0.001**	**(0.344–0.488)**	**0.647**	**<0.001**	**(0.537–0.757)**
6 months vs. 1 month (ref)	−0.164	0.478	(−0.616–0.288)	−0.472	0.935	(−11.780–10.854)
**Types of LV remodeling**						
Adverse	Ref	Ref	Ref	Ref	Ref	Ref
Reverse ^a^	**1.093**	**<0.001**	**(0.613–1.573)**	**41.448**	**<0.001**	**(30.363–52.533)**
Reverse ^b^	**1.122**	**<0.001**	**(0.640–1.604)**	**40.698**	**<0.001**	**(28.786–52.611)**

Footnotes for Table 3: ^a^ Corrected for changes in protein levels; ^b^ Corrected for changes in protein levels, age, gender, race, medical history, lipid levels at baseline and medication. Bolded values are those with *p*-value < 0.05.

**Table 4:** Area under the receiver-operating curve (AUC) of different candidate markers predicting reverse LV remodeling.

Biomarker	Predicting Reverse LV Remodeling		
	AUC	95% CI	*p*-Value
NT-proBNP	0.384	0.305–0.463	0.005
hsTnI	0.467	0.386–0.548	0.419
hsTnT	0.389	0.311–0.467	0.007
VWF:Plasminogen	0.674	0.599–0.748	<0.001
SerpinC1:Plasminogen	0.712	0.639–0.786	<0.001
NT-proBNP + hsTnI + hsTnT	0.628	0.550–0.706	0.002
VWF:Plasminogen + SerpinC1:Plasminogen	0.717	0.645–0.790	<0.001
All 5 biomarkers	0.763	0.697–0.829	<0.001

## Data Availability

The data presented in this study are available on request from the corresponding author.

## Supplementary Materials

The following supporting information can be downloaded at: https://www.mdpi.com/article/10.3390/ijms24010327/s1, Figure S1: Characterization of LDL-EVs of post-AMI patients with adverse and reverse LV remodeling; Table S1. Baseline and follow-up LDL-EVs protein levels in patients.
