# Supplementary material for: Temporal Changes in Extracellular Vesicle Hemostatic Protein Composition Predict Favourable Left Ventricular Remodeling after Acute Myocardial Infarction
Source: Int J Mol Sci. 2022 Dec 25;24(1):327. doi: 10.3390/ijms24010327 (PMC9820565; doi:10.3390/ijms24010327)
Supplement: Supplementary file 1 [file ijms-24-00327-s001.zip › ijms-2049033-supplementary.pdf]

International Journal of *Molecular Sciences*

MDPI

Supplemental Data

Temporal changes in extracellular vesicle hemostatic protein composition predict favourable left ventricular remodeling after acute myocardial infarction

Xiong Chang Lim[1], Chenyuan Huang[1,2], Siti Maryam J.M. Yatim[1,2], Suet Yen Chong[1,2], Sock Hwee Tan[2,3], Xiaoxun Yang[2,3], Caryn L. Heldt[4], Jodi Pedersen[4], Michael Talanker[4], Harshvardhan Modh[5], Matthias G. Wacker[5], Giorgia Pastorin[5], Siew Pang Chan[2,3], A. Mark Richards[2,3,6], Chris J. Charles[1,2,6], Mark Y. Chan[2,3,*], Jiong-Wei Wang[1,2,7,8,*]

[1] Department of Surgery, Yong Loo Lin School of Medicine, National University of Singapore, Singapore, Singapore
[2] Cardiovascular Research Institute (CVRI), National University Heart Centre Singapore (NUHCS), Singapore, Singapore
[3] Department of Medicine, Yong Loo Lin School of Medicine, National University of Singapore, Singapore, Singapore
[4] Department of Chemical Engineering, Michigan Technological University, Houghton, Michigan, USA
[5] Department of Pharmacy, Faculty of Science, National University of Singapore, Singapore, Singapore
[6] Christchurch Heart Institute, Department of Medicine, University of Otago, Christchurch, New Zealand
[7] Department of Physiology, Yong Loo Lin School of Medicine, National University of Singapore, Singapore, Singapore
[8] Nanomedicine Translational Research Programme, Centre for NanoMedicine, Yong Loo Lin School of Medicine, National University of Singapore, Singapore, Singapore
* Correspondence: mark_chan@nuhs.edu.sg (M.Y.C.); surwang@nus.edu.sg (J.-W.W.)

Supplemental Tables

Table S1. Baseline and follow-up LDL-EVs protein levels in patients

LDL-EVs Protein Levels [log (pg/mL)	Post-AMI					
	Adverse LV Remodeling n = 98			Reverse LV Remodeling n = 100		
	Baseline	1 month	6 months	Baseline	1 month	6 months
**Coagulation Protein**						
VWF	14.32 (13.70-14.94)	13.99 (13.33-14.50)	13.77 (13.26-14.18)	14.44 (13.91-15.04)	14.09 (13.64-14.68)	14.26 (13.63-14.66)
SerpinC1	17.79 (17.52-18.16)	17.67 (17.46-18.08)	17.66 (17.38-18.06)	17.79 (17.54-18.23)	17.81 (17.55-18.17)	17.85 (17.63-18.22)
**Fibrinolytic Protein**						
Plasminogen	13.68 (13.32-13.99)	13.85 (13.45-14.19)	13.89 (13.49-14.29)	13.56 (13.11-14.12)	13.32 (12.89-13.92)	13.36 (12.98-14.10)

Data are presented as median ± IQR. Abbreviation: LDL, low density lipoprotein; EV, extracellular vesicles; AMI, acute myocardial infarction.

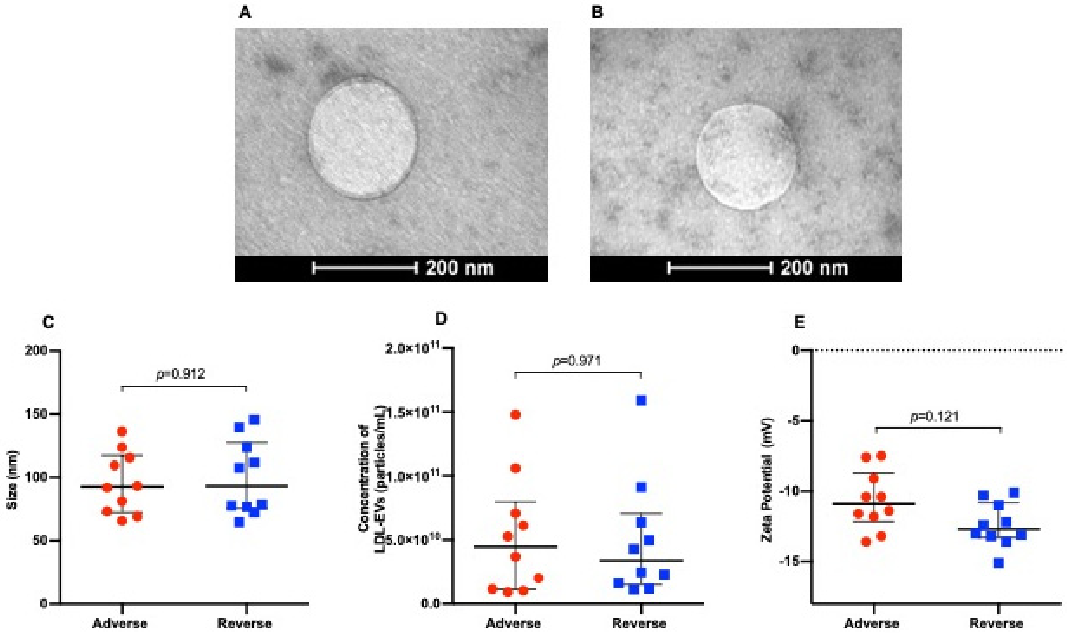

Figure S1. Characterization of LDL-EVs of post-AMI patients with adverse and reverse LV remodeling. A: TEM image of LDL-EV from patient with adverse LV remodeling. **B:** TEM image of LDL-EV from patient with reverse LV remodeling. **C, D, E:** Size, concentration, and zeta potential of LDL-EVs of 20 post-AMI patients (n=10 adverse LV remodeling and n=10 reverse LV remodeling) at baseline. Differences in characteristics between patients with adverse LV remodeling and reverse LV remodeling were established by Mann-Whitney U test.
